# Realization of a deeply subwavelength adiabatic optical lattice

**DOI:** 10.1103/physrevresearch.2.013149

**Published:** 2020

**Authors:** R. P. Anderson, D. Trypogeorgos, A. Valdés-Curiel, Q.-Y. Liang, J. Tao, M. Zhao, T. Andrijauskas, G. Juzeliūnas, I. B. Spielman

**Affiliations:** 1 Joint Quantum Institute, University of Maryland, College Park, Maryland 20742, USA; 2 School of Physics and Astronomy, Monash University, Melbourne, Victoria 3800, Australia; 3 La Trobe Institute of Molecular Science, La Trobe University, Bendigo, Victoria 3552, Australia; 4 INO-CNR BEC Center and Dipartimento di Fisica, Università di Trento, 38123 Povo, Italy; 5 Institute of Theoretical Physics and Astronomy, Vilnius University, Saulėtekio 3, LT-10257 Vilnius, Lithuania; 6 National Institute of Standards and Technology, Gaithersburg, Maryland 20899, USA

## Abstract

We propose and describe our realization of a deeply subwavelength optical lattice for ultracold neutral atoms using *N* resonantly Raman-coupled internal degrees of freedom. Although counterpropagating lasers with wavelength *λ* provided two-photon Raman coupling, the resultant lattice period was *λ*/2*N*, an *N*-fold reduction as compared to the conventional *λ*/2 lattice period. We experimentally demonstrated this lattice built from the three *F* = 1 Zeeman states of a ^87^Rb Bose-Einstein condensate, and generated a lattice with a *λ*/6 = 132 nm period from *λ* = 790 nm lasers. Lastly, we show that adding an additional rf-coupling field converts this lattice into a superlattice with *N* wells uniformly spaced within the original *λ*/2 unit cell.

## I. INTRODUCTION

Optical lattices form a vital substrate for quantum gases, enabling the quantum simulation of iconic condensed matter systems [1], the realization of new atomic topological materials [2], and new-generation atomic clocks [3]. Generally the spatial period of an optical lattice is derived from the difference of the wave vectors of the underlying laser beams (with wavelength *λ*), forging an apparent lower limit of *λ*/2 to the lattice period. Many techniques can add subwavelength structure to a lattice, ranging from Raman methods [4], radio-frequency-dressed state-dependent optical lattices [5,6], and time-modulated “Floquet” lattices [7], to deeply subwavelength structures using dark states [8–10]. Going beyond these techniques, only explicit use of multiphoton transitions has to date reduced the underlying lattice period in quantum-gas experiments [11–13]. In addition to increasing the overall energy scales, potentially easing the preparation of low-temperature states, each of these short-period optical lattices offers new experimental opportunities: changing the balance between on-site and nearest-neighbor interactions [8–10]; sculpting potential at a subwavelength scale [11–13]; and engineering artificial gauge fields [7]. Here, we propose and demonstrate a flexible subwavelength lattice with period *λ*/2*N* built from *N* resonantly coupled atomic states; furthermore, an additional coupling field converts the lattice to a tunable superlattice with *N* wells arrayed within a conventional *λ*/2 unit cell.

Any 1D lattice can be described by a Hamiltonian $\hat{H}(\hat{x})=\hat{H}(\hat{x}+\delta x)$ that is invariant under spatial displacements *δx* [14]. The smallest such displacement *d* defines the lattice’s unit cell, and correspondingly $\hat{H}(\hat{x})$ couples momentum states differing by integer multiples of the resulting reciprocal lattice constant *k*_0_ = 2*π*/*d*. An optical lattice with spatial period *λ*/2 formed by a pair of counterpropagating lasers with wavelength *λ* and single-photon momentum *ħk*_*R*_ = 2*πħ*/*λ* is intuitively derived from the *ħk*_0_ = 2*ħk*_*R*_ momentum obtained by exchanging photons between lattice lasers. This 2-photon concept has been directly extended to higher-order 4- or even 6-photon transitions producing lattices with reduced periods [11–13], but required a concomitant increase in laser intensities, giving greatly reduced lifetimes as compared to 2-photon lattices generated from lasers with smaller *λ*. In this work, we exploit a gauge symmetry present for *N* internal atomic states coupled by conventional two-photon Raman transitions to generate a highly tunable lattice with period *λ*/2*N*.

## II. PROPOSAL

**FIG. 1.**
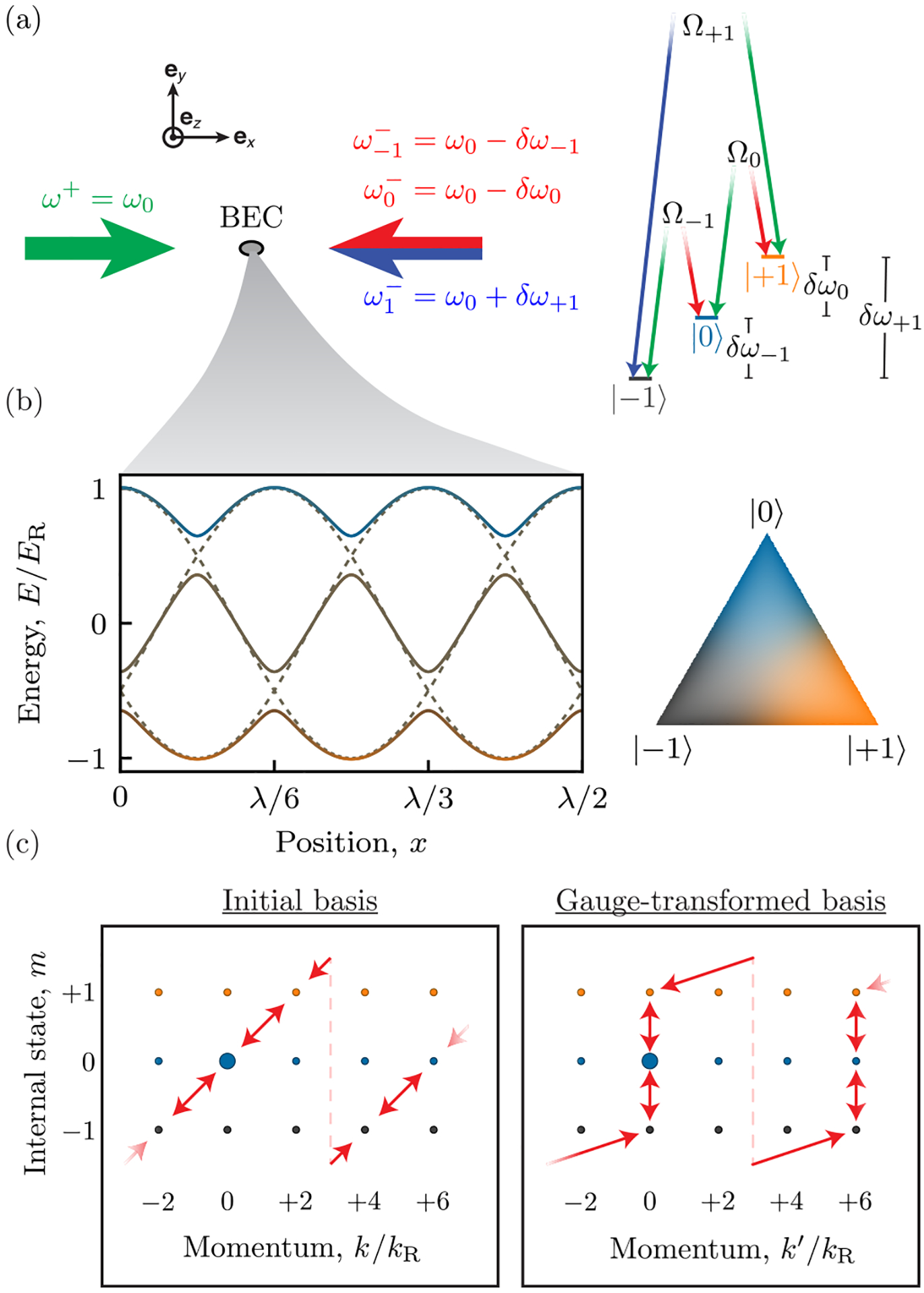
Experimental and conceptional schematic for *N* = 3. (a) Experimental geometry and level diagram. A BEC is illuminated by a pair of laser beams that complete two-photon Raman transitions between *N* internal states. (b) Computed adiabatic potentials illustrating the spatial subdivision of this lattice. The dashed curves were computed for *ħ***Ω** = (1.0, 1.0, 1.0) × *E*_*R*_ and ***δ*** = **0**; the solid curves were computed for *ħ***Ω** = (1.0, 1.25, 0.75) × *E*_*R*_ and ***δ*** = **0**. The ternary diagram to the right shows the color scheme used throughout this paper to indicate the fractional composition of states. (c) Internal state–momentum coupling diagrams showing nodes describing states (labeled by internal state and momentum) connected by links denoting the laser-induced momentum kicks on each link (left) transferred to a single link (right) after the state-dependent gauge transformation.

Our lattice derives from *N* cyclically coupled internal atomic states [15] labeled by |*m*〉, using an angular-momentum-inspired convention with *m* ranging from −*g* to +*g*, with *g* = (*N* − 1)/2, and with a periodic convention |*N* + *m*〉 = |*m*〉. The two-photon Raman transitions [shown in Fig. 1(a) for the *N* = 3 case] from lasers counterpropagating along **e**_*x*_ couple consecutive states with matrix element $\left[\hbar \Omega_{m}\text{exp}\left(2ik_{R}\hat{x}\right)/2\right]|m+1\rangle \langle m|$. The resultant light-matter interaction term

(1) $$\frac{\hat{V}(\hat{x})}{\hbar }=\sum_{m}\frac{\Omega_{m}}{2}e^{2ik_{R}\hat{x}}|m+1\rangle \langle m|+\text{ H}.\text{c}.\;,$$

and state-dependent energy shifts $\hat{\Delta }/\hbar =\sum_{m}\delta_{m}|m\rangle \langle m|$ are manifestly invariant under discrete spatial translations by multiples of *λ*/2. This lattice’s true nature is evidenced by the adiabatic potentials resulting from diagonalizing $\hat{V}(\hat{x})+\hat{\Delta }$, as plotted in Fig. 1(b) for *N* = 3. These potentials repeat three times within the purported *λ*/2 unit cell, suggesting an *N*-fold reduced unit cell, and therefore an *N*-fold enlarged Brillouin zone (BZ). This reduction is made explicit by a state-dependent gauge transformation $\hat{\Phi }(\hat{x})=\text{exp}\left(i\sum_{m}2mk_{R}\hat{x}|m\rangle \langle m|\right)$ that leaves $\hat{\Delta }$ unchanged, but takes $\hat{V}(\hat{x})$ into

$$\frac{{\hat{V}}^{\prime }(\hat{x})}{\hbar }=\left(\frac{\Omega_{g}}{2}e^{2iNk_{R}\hat{x}}|-g\rangle \langle g|+\sum_{m=-g}^{g-1}\frac{\Omega_{m}}{2}|m+1\rangle \langle m|\right)+\text{ H}.\text{c}.\;,$$

revealing a unit cell [16] with size *d* = *λ*/2*N* and reciprocal lattice vector *k*_0_ = 2*k*_*R*_*N* [17]. Figure 1(c) compares the transitions driven by $\hat{V}(\hat{x})$ (left) with those possible from ${\hat{V}}^{\prime }(\hat{x})$ (left), in both cases starting in |*k* = 0, *m* = 0〉 for *N* = 3. This shows that $\hat{V}(\hat{x})$ couples states of all momenta separated by 2*k*_*R*_, albeit in different internal states, while ${\hat{V}}^{\prime }(\hat{x})$ either changes the momentum in units of 6*k*_*R*_ or leaves it unchanged. Similarly to 1D spin-orbit coupling experiments [18], this gauge transformation also introduces a spatially uniform vector-gauge potential $\hat{A}=-\sum_{m}2\hbar k_{R}m|m\rangle \langle m|$ leading to the kinetic energy ${\left(\hbar {\hat{k}}^{\prime }-\hat{A}\right)}^{2}/2M$; we denote the momentum in the gauge-transformed frame as *ħk*′. During the preparation of this paper, we learned of a proposal [19] that focuses on a similar lattice for ring-shaped traps using Laguerre-Gauss Raman laser modes, but which notes the connection to linear geometries.

The dashed curves in Fig. 1(b) depict an additional feature of this system: in the special case of zero detuning $\hat{\Delta }=0$ and isotropic coupling Ω_*i*_ = Ω_*j*_, the subwavelength lattice fragments into three interwoven *d* = *λ*/2 sinusoidal lattices, each comprised of equally weighted superpositions of the |*m*〉 states.

## III. IMPLEMENTATION

Our experiments began with nearly pure ^87^Rb BECs in a crossed optical dipole trap [20], with frequencies (*f*_*x*_, *f*_*y*_, *f*_*z*_) ≈ (45, 55, 160) Hz. A magnetic field *B*_0_ = 3.4031(1)mT along **e**_*z*_ Zeeman-split the |*m*_*F*_ = −1, 0, +1〉 states comprising the ground state *F* = 1 manifold by *ω*_*Z*_/2*π* ≈ 23.9MHz. We first coupled these states with strength Ω_rf_/2*π* = 134.5(1)kHz, using a strong radio frequency (rf) magnetic field oscillating along **e**_*x*_ with angular frequency *ω*_*Z*_. This made a trio of dressed states [21–23] which we denote by |*m* = −1〉, |0〉, and |+1〉 with energies *ħω*_±1,0_. Unlike the bare *m*_*F*_ states, every pair of these states can be Raman coupled using lasers detuned far from resonance as compared to the excited-state hyperfine splitting [21]. The resulting three energy splittings (*δω*_−1_, *δω*_0_, *δω*_+1_)/2*π* = (182.4(1),99.2(1),281.6(1))kHz with *δω*_*m*_ = |*ω*_*m*+1_ − *ω*_*m*_| were at least first-order insensitive to magnetic field fluctuations, rendering our experiment insensitive to the 0.1 *μ*T scale magnetic field noise in our laboratory. These states are further immune to the 2-body spin relaxation collisions that often plague mixtures between different hyperfine manifolds.

We coupled the dressed states using a pair of cross-polarized Raman laser beams counterpropagating along **e**_*x*_ tuned to the “magic-zero” wavelength *λ* = 790 nm where the scalar light shift vanishes [24]. This laser served to define the single-photon recoil energy $E_{R}=\hbar^{2}k_{R}^{2}/2M$ for atoms with mass *M*. The Raman beam traveling along +**e**_*x*_ had frequency [25] *ω*^+^ = *ω*_0_ + *ω*_*Z*_, while the beam traveling along −**e**_*x*_ carried frequencies $\omega_{m}^{-}=\omega_{0}\pm \left(\delta \omega_{m}+\delta_{m}\right)$. In what follows we maintain the detunings *δ*_*j*_ ≈ 0 to within our experimental control, and state their measured values in the figure captions. The ± is selected as indicated in Fig. 1(a), such that |−*g*〉 to |+*g*〉 transition has the opposite frequency shift to impart the same phase factor as the remaining transitions. Changing the sign on any *δω*_*m*_ leaves the transition in Raman resonance, but inverts the sign of the associated phase factor in Eq. (1), increasing the unit cell size as noted above. The coupling strength of each Raman transition *ħ*Ω_*m*_ ≲ *E*_*R*_ from state |*m*〉 to |*m* + 1〉 was far smaller than the *δω*_*m*_ spacing between the three |*m*〉 states. This simultaneously ensured the validity of the rotating-wave approximation and rendered negligible second-order energy shifts due to off-resonant coupling to other transitions.

In the following experiments, we prepared the BEC in any of the three |*m*〉 states [22] before applying any additional coupling fields. We measured the final state by first abruptly turning off the Raman lasers and the dipole trap, thereby projecting onto the |*m*〉 states and free momentum states. We then adiabatically transformed the |*m*〉 states back to the standard |*m*_*F*_〉 spin states in about 1ms by simultaneously ramping *B*_0_ away from rf resonance and ramping Ω_rf_ to zero [22]. During the following 21 ms time-of-flight the Stern-Gerlach force from a magnetic field gradient first separated these spin states, and we obtained the resulting spin-resolved momentum distribution using absorption imaging.

## IV. ENLARGED BZ

We experimentally resolved the enlarged BZ by measuring the internal-state composition in the lowest band as a function of the crystal momentum *ħq* and show that it repeats every 6*ħk*_*R*_ rather than every 2*ħk*_*R*_ as would be expected for a λ/2 lattice period. This shows that the BZ must be at least 6*ħk*_*R*_ in extent, but does not exclude the possibility of a BZ even larger than predicted. Given the agreement of our experiment with our microscopic model, we take this as support of our model which contains a 3-fold enlarged BZ.

**FIG. 2.**
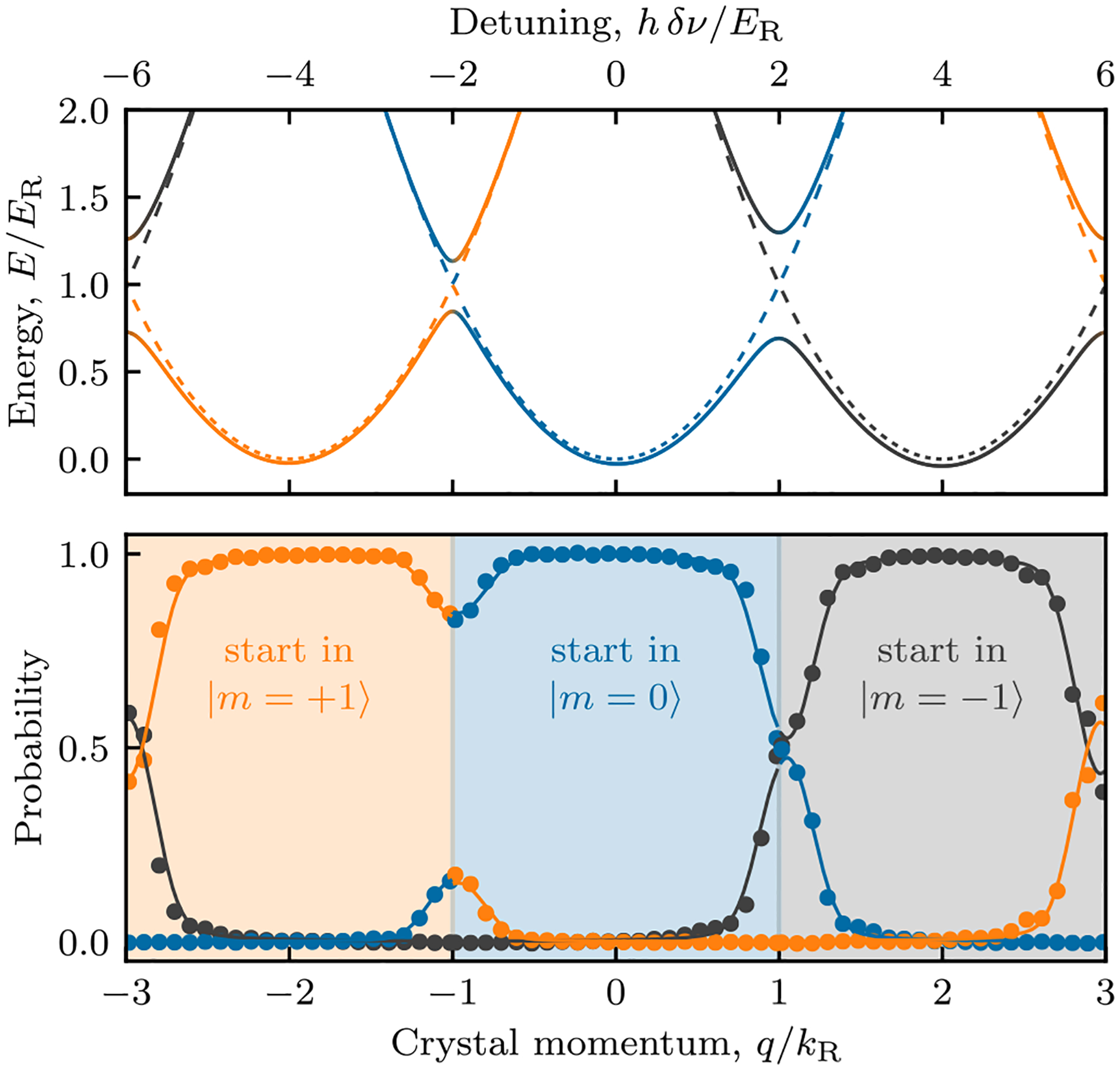
Enlarged BZ. The top axis shows the frequency shift *δν* used to effect a moving lattice that populated the desired *ħq* state. Top: Computed band structure. Each solid curve is shaded in accordance with the population in the |−1〉, |0〉, |+1〉, using the ternary diagram in Fig. 1(b). The dashed curves depict the bare free particle dispersion absent Raman coupling. Bottom: Internal state composition of the lowest band. Different regions of the BZ were explored by starting in the three |*m*〉 states. The solid curves plot the outcome of a full simulation of our experimental protocol. We determine the Raman coupling strengths to be *ħ***Ω** = (0.54(1), 0.29(1), 0.61(1)) × *E*_*R*_ and detunings *ħ*|*δ*_*m*_| ⩽ 0.07(1)*E*_*R*_.

We used the narrow momentum distribution of a BEC to probe individual Bloch states, and rather than accelerating the BEC to access nonzero crystal momentum states, we instead ramped on a moving lattice in 200 *μ*s, adiabatically loading BECs at rest in the laboratory frame into nonzero crystal momentum states of the moving lattice. We brought the lattice into motion [26,27] by detuning one of the two Raman lasers by *δν*, giving a crystal momentum of *q*/*k*_*R*_ = *h δν*/(4*E*_*R*_) in the lattice’s rest frame. After a brief 50 *μ*s hold in the moving lattice, we measured the state-resolved momentum distribution. As shown in Fig. 2 (top), the lowest band contains three local minima near *q* = −2*k*_*R*_, 0, or 2*k*_*R*_, predominantly derived from the |+1〉, |0〉, or |−1〉 state, respectively. The first excited band approaches the lowest band at band gaps (avoided crossings) between these minima, rendering our lattice turn-on nonadiabatic in their vicinity. Accordingly, we accessed the entirety of the enlarged BZ in a piecewise manner: for each of the three initial states, we applied the above method to focus on a single 2*ħk*_*R*_ interval.

We operated in a regime of unequal coupling (see caption), where the adiabatic potential cannot be decomposed into independent sinusoids. Figure 2 (bottom) shows the measured occupation probability in each of the |*m*〉 states, immediately exposing the enlarged BZ. The solid curves are the result of a numerical simulation via unitary dynamics of our loading procedure which are in near-perfect agreement with our measurements. The differing population ratios in the vicinity of the band gaps result from the asymmetric Raman coupling.

## V. SUPERLATTICE

We conclude by describing how to gain individual control over the energy minima of the *N* sublattice sites discussed above, essentially combining *N* reduced unit cells into a superlattice structure: a *λ*/2 period lattice with *N* basis states (sites). We demonstrate this principle by creating a tunable triple-well lattice.

**FIG. 3.**
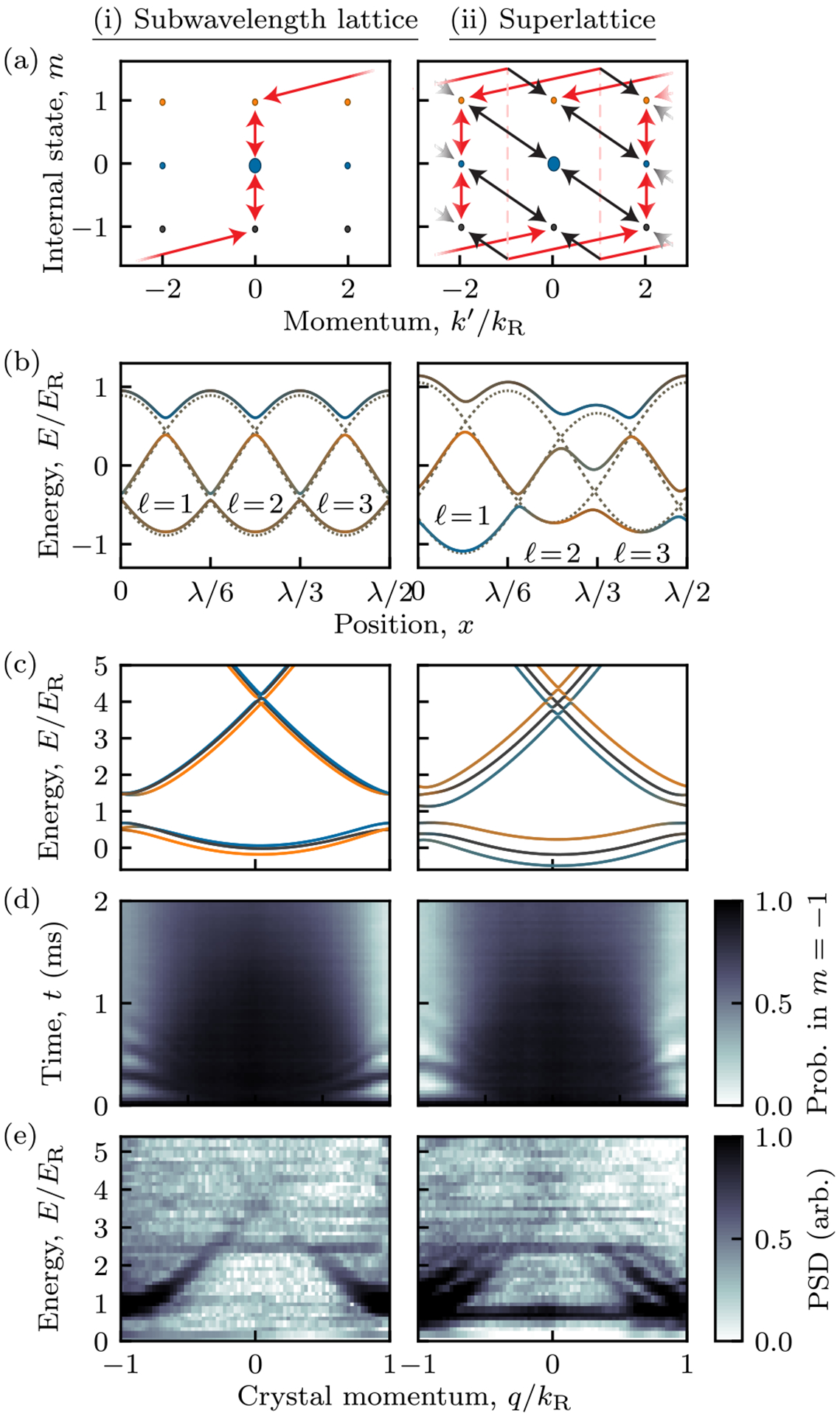
(i) Subwavelength lattice and (ii) superlattice. (a) Momentum-coupling graphs as in Fig. 1(c), with red links from Raman coupling and black links from rf coupling. Panels (b)–(e) share the same parameters with *ħ***Ω** = (0.87, 0.98, 0.82) × *E*_*R*_ and *ħ****δ*** = (0.08, 0.08, 0.15) × *E*_*R*_, with columns (i) and (ii) depicting *ħ***Ω**_rf_ = **0** and *ħ***Ω**_rf_ = (0, 0.7, 0) × *E*_*R*_, respectively. (b) Computed adiabatic potentials with rf phase *ϕ*_0_ = *π*/4. (c) Computed band structure for the experimental parameters [shaded per Fig. 1(b)]. (d) Measured probability in |−1〉 as a function of time and crystal momentum, for a system initially in |−1〉. Dark and light color tones indicate high and low probability, respectively. (e) PSDs obtained from time traces such as in (c). Each PSD is derived from the time evolution of all three internal states, averaged over five time series. For the Raman-only data (i) we started in the |*m* = −1〉 state only, while for the Raman and rf data in (ii) we increased the signal-to-noise ratio by combining data starting in all three internal states. The horizontal structures present in both data sets around 2.5*E*_*R*_ result from laboratory technical noise.

We break the symmetry between the three sublattice sites using a spatially homogeneous coupling with strength $\Omega_{m}^{\text{rf}}$ and phase $\phi_{m}^{\text{rf}}$ to the matrix element in Eq. (1), giving combined matrix elements such as $\left[\Omega_{m}\text{exp}\left(2ik_{R}\hat{x}\right)+\Omega_{m}^{\text{rf}}\text{exp}\left(i\phi_{m}^{\text{rf}}\right)\right]/2$. Figure 3(a) shows a connected graph with nodes depicting the collection of momentum and internal states that a system initially in |*k*′ = 0, *m* = 0〉 can couple to, with red and black links denoting Raman and rf transition matrix elements, respectively. In the gauge-transformed frame all rf transitions change momentum [are diagonal in Fig. 3(a)], while only some Raman transitions change momentum. Figures 3(a)–3(i), with only Raman coupling, are simply an expanded view of Fig. 1(c), where the coupled states differ in momentum by 6*ħk*_*R*_, yielding a *λ*/6 period, but in (ii) the rf term couples to the previously disconnected groups of states differing in momentum by just 2*ħk*_*R*_, yielding a *λ*/2 period. The most significant effect of even one such rf coupling is to shift the energies within the unit cell as shown in Fig. 3(b). As in Fig. 1(b), the dashed curves show the three uncoupled sinusoids present for uniform detuning and Raman coupling, which we now enumerate with *ℓ*, ranging from 1 to *N*. At lowest order rf shifts these curves by an energy $\hbar \Omega_{m}^{\text{rf}}\text{cos}\left(2\pi \ell /N+\phi_{m}^{\text{rf}}\right)/N$, and at higher order it introduces new transition matrix elements between the adiabatic potentials. Figure 3(c) shows the resulting band structure in the initial BZ. In the subwavelength lattice case (i), the single lowest band appears as a group of three bands, which are connected across the edge of the BZ; in the superlattice case (ii), this single connected curve becomes gapped at the edge of the BZ, splitting into three distinct bands. Just as the familiar bipartite (double-well) Su-Schrieffer-Heeger model [28] has a pair of low-energy bands, this *N*-partite lattice has *N* low-energy bands.

We experimentally demonstrated this concept by adding one rf-coupling field to our Raman lattice and directly verified the formation of the superlattice potential using Fourier transform spectroscopy [29], which we detail in Appendix A. The essential concept of this technique is that as a quantum system undergoes unitary evolution, the observed probabilities have a spectrum composed of all the frequency differences between the energy eigenstates, with amplitude proportional to the transition matrix elements. The frequency differences are obtained from the power spectral density (PSD; magnitude-squared of the Fourier transform) of experimental time traces. Here we apply this technique starting with noncondensed ensembles [30] spanning the whole BZ, and in any internal state; we simultaneously applied the Raman coupling and a single rf coupling $\Omega_{0}^{\text{rf}}$, coupling |*m* = 0〉 with |*m* = +1〉. We then measured the resulting time-evolving state-resolved momentum distributions for 2 ms. Figure 3(d) shows representative momentum distributions spanning the BZ as a function of time for atoms starting and ending in internal state |*m* = −1〉.

Figure 3(e) shows the PSDs computed for each initial momentum state separately, both without (left) and with (right) rf coupling, with a ≈0.5 kHz frequency resolution from the 2ms evolution time. The Raman-only data (i) are dominated by a single difference frequency associated with the Raman lattice’s splitting; here the nearly degenerate spectra associated with the enlarged BZ lie atop each other and cannot be distinguished. The addition of rf coupling (ii) lifted this degeneracy and produced three subbands, each associated with a single site of the 3-partite lattice, which are manifested by the appearance of three resolvable energy differences in the Fourier transform spectrum.

## VI. CONNECTION TO SINUSOIDAL LATTICES

**FIG. 4.**
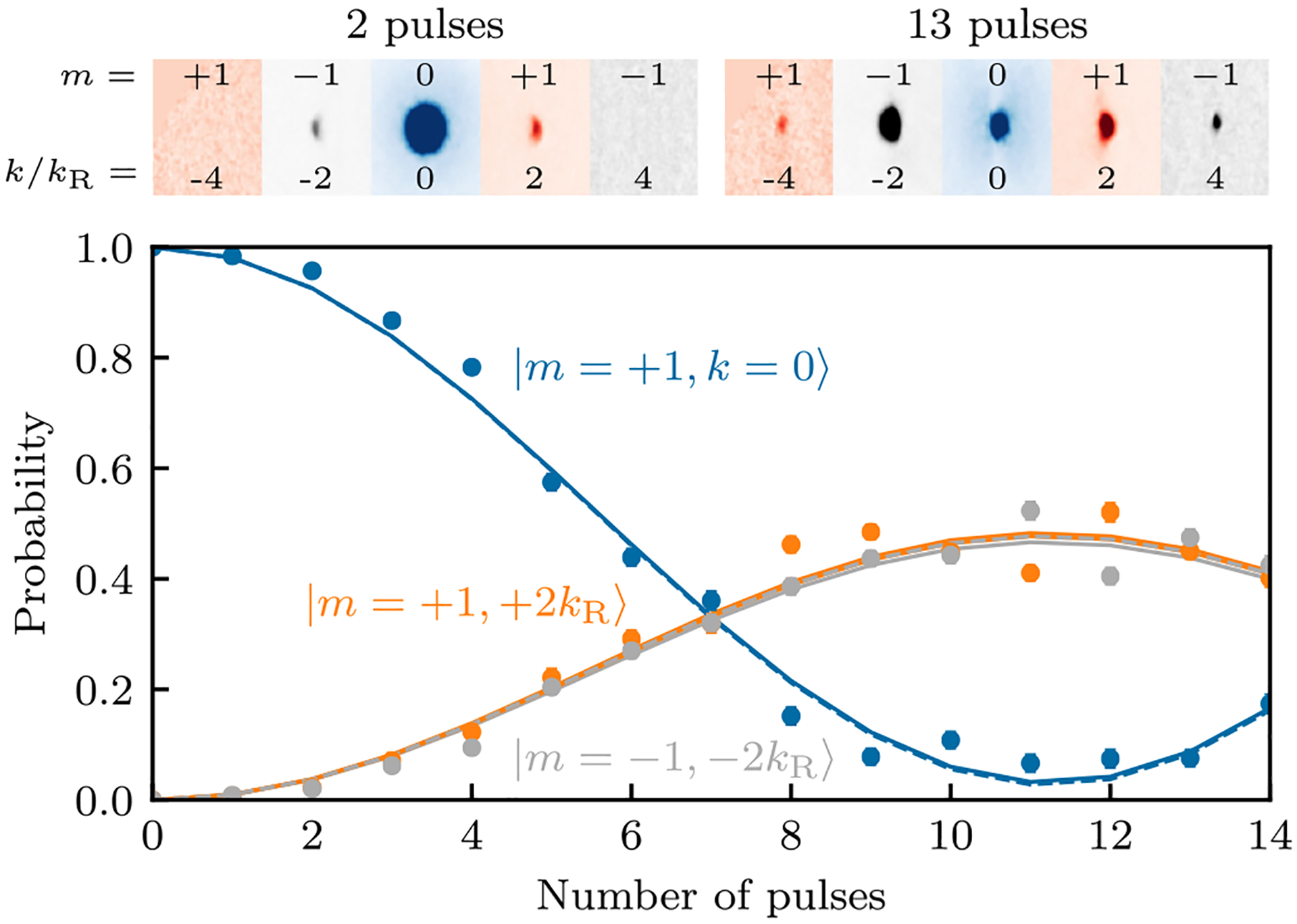
Stroboscopic evolution starting in |*m* = 0〉. During each pulse the Raman lasers were applied for 50 *μ*s and then removed for 16.8 *μ*s, giving a total pulse duration close to *h*/(4*E*_*R*_). Top: Absorption images showing the initial *k* = 0 state and diffracted orders for 2 and 13 pulses, respectively. The symbols plot the population |*m* = 0〉, |+1〉, and |−1〉, colored blue, orange, and gray, respectively. Bottom: The solid curves depict our full lattice model using calibrated couplings *ħ***Ω** = (0.58(1), 0.57(1), 0.58(1)) × *E*_*R*_ and detunings *ħ****δ*** = (0, −0.03(1), 0) × *E*_*R*_. The detuning of the initial state *δ*_0_ was the only fit parameter. The dashed curve plots the prediction of the simple lattice model with a depth *ħ*(Ω_0_ + Ω_−1_) = 1.15(2)*E*_*R*_ and the same −0.03*E*_*R*_ energy shift of the initial state.

The three gray dashed adiabatic potentials shown in Fig. 1(b) rightly suggest that in the simple case of uniform coupling $\Omega_{m}=\bar{\Omega }$ and zero detuning ***δ*** = **0**, the lattice decomposes into three independent sinusoidal lattices each with depth $2\hbar \bar{\Omega }$ obtained by diagonalizing ${\hat{V}}^{\prime }(\hat{x})$. We confirmed this picture by following the unitary evolution of a BEC suddenly exposed to all three Raman fields simultaneously and observed diffraction into discrete momentum orders spaced by 6*ħk*_*R*_ within each final internal state offset by ±2*ħk*_*R*_ in the |*m* = +1〉 and |*m* = −1〉 states, respectively, as shown in the top panel of Fig. 4. For any initial internal state, the dynamics of these orders individually were indistinguishable from the 2*ħk*_*R*_*n* orders diffracting off a conventional 1D optical lattice.

We enhanced the diffraction from our comparatively shallow lattice by pulsing it repeatedly [31]: alternating between periods of evolution with and without the lattice present allows a state initially in |*k* = 0〉 to acquire far more population in |*k* = ±2*k*_*R*_〉 than from a single uninterrupted pulse of any duration. Figure 4 shows the resulting evolution for a system initially in |*m* = 0〉; the solid curves depict the prediction of our full lattice model using independently calibrated couplings (see caption). For weak Raman coupling such as ours, the matrix elements directly coupling the initial state dominate the dynamics. The dashed curves depict the prediction of a simple 1D lattice with depth *ħ*(Ω_*m*=0_ + Ω_*m*=−1_) = 1.15(2)*E*_*R*_.

## VII. TIGHT-BINDING MODEL

The general band structure of this lattice must be obtained through numerical diagonalization. For zero detuning and nearly equal couplings $\Omega_{m}=\bar{\Omega }+\delta \Omega_{m}$, with $\left|\delta \Omega_{m}\right|\ll \bar{\Omega }$, some lattice properties reduce to those of the conventional *λ*/2 lattice [32] of depth $sE_{R}=2\hbar \bar{\Omega }$ described in the previous section. For example, the gap between the lowest two bands is *sE*_*R*_/2 for *s* ≪ 4 and $(2\sqrt{s}-1)E_{R}$ for *s* ≫ 4.

More specifically, the tight-binding model suitable for the ground band of this lattice consists of *N* sites within each original unit cell derived from the *N* minima of the adiabatic potentials in Fig. 1(b) (gray dashed curves). Each minima is associated with a Wannier orbital *W* (*x*) well approximated by a Gaussian with width *w* = *λ*/(2*πs*^1/4^) centered at *x* = *j* × *λ*/(2*N*). (We note that the Gaussian approximation becomes poor outside the lattice site it resides in, i.e., a range of about ±*λ*/4, beyond which numerical methods are required.) Following Ref. [33], this adds long-range tunneling terms

$$J_{j,\delta j}^{\prime }=\frac{\delta {\tilde{\Omega }}_{\delta j}}{2N^{1/2}}\text{exp}\left[-\frac{1}{\sqrt{s}}\right]\text{exp}\left[-\frac{\sqrt{s}}{4}{\left(\frac{\pi \delta j}{N}\right)}^{2}\right]\times \text{exp}\left[-\frac{i\pi (\delta j+2j)}{N}\right]$$

describing the tunneling amplitude from site *j* to site *j* + *δ j*. The terms in this expression are interpreted as follows. The first term $\delta {\tilde{\Omega }}_{\delta j}$, the discrete Fourier transform of the differences *δ*Ω_*m*_ sets, the scale of the added tunneling of range *δ j*. The second term is a Lamb-Dicke suppression term which becomes negligible for large *s*, where the Wannier function becomes small compared to the optical wavelength. The third term captures the spatial overlap of Wannier orbitals separated by *δ j*. The final term is a Peierls phase from the $\text{exp}\left(2ik_{R}\hat{x}\right)$ coupling matrix elements.

For a lattice of depth *s* = 5 with *N* = 3 and $\delta {\tilde{\Omega }}_{1}=1E_{R}$, this gives significant nearest-neighbor tunneling $\left|J_{1}^{\prime }\right|\approx 0.05E_{R}$, minimal next-nearest-neighbor tunneling $\left|J_{2}^{\prime }\right|\approx 0.008E_{R}$, along with the range *δ j* = 3 hopping *J* = 0.07*E*_*R*_ from the *λ*/2 adiabatic potentials.

Lastly, assuming a 1D system with contact interactions with strength *g*, the model acquires non-negligible long-range Hubbard terms

$$\begin{array}{l} U_{\delta j}=g\int dx\left|w(x){|}^{2}\right|w(x+\delta j\lambda /2N){|}^{2}\\ =U\times \text{exp}\left[-\frac{\sqrt{s}}{2}{\left(\frac{\pi \delta j}{N}\right)}^{2}\right], \end{array}$$

where *U* ≡ *U*_0_ is the standard on-site Hubbard *U*. For a lattice of depth *s* = 5 and with *N* = 3, this gives significant nearest-neighbor interactions *U*_1_/*U* ≈ 0.3, but small next-nearest-neighbor interactions *U*_2_/*U* ≈ 0.01, an ideal starting point for experiments searching for supersolid or density-wave phases [34].

All together this shows that for a typical *U* ≈ 1.5*E*_*R*_, our lattice as realized was already in the single-band regime, and similarly to the lattice proposed in Ref. [35], the resulting Hubbard system produces a highly tunable 1D lattice both in terms of the single-particle tunneling terms and the interaction terms.

## VIII. OUTLOOK

The ≈1 ms lifetime for coherent evolution in our experiment resulted from interaction effects common when excited Bloch bands are populated [36]; in contrast the spontaneous-emission-limited lifetime is computed and measured to be hundreds of milliseconds (see Appendix B).

Our techniques are readily extendable to higher spatial dimensions [37]: in 2D, kagome lattices can be generated using the same three internal states used here, and in 3D, pyrochlore-type lattices can be assembled using four internal states. The latter are of particular interest as they are a candidate lattice for realizing non-Abelian topological spin and charge pumps [38] derived from the second Chern number [39]. The specific three-site superlattice demonstrated here is ideally suited for assembling gauge fields without spatial gradients [40]. In the broader setting, subwavelength optical structures may defeat the diffraction limit for lithography, using either nonclassical light [41] or even coherent atomic dynamics [42].

## APPENDIX A: FOURIER TRANSFORM SPECTROSCOPY

Fourier transform spectroscopy is a technique used for measuring single-particle energies that relies on measuring the quantum-coherent evolution of a system subject to a quench of a Hamiltonian of interest *Ĥ*. We consider a set of orthonormal states {|*ψ*_*i*_〉} that fully span the accessible Hilbert space of the system and whose occupation probabilities are experimentally measurable. The time evolution of an arbitrary initial sate |Ψ_0_〉 = Σ_*i*_ *a*_*i*_ |*ψ*_*i*_〉 suddenly subjected to *Ĥ* is $|\Psi (t)\rangle =\sum_{i,j}a_{i}c_{i,j}e^{-iE_{j}^{\prime }t/\hbar }|\psi_{j}^{\prime }\rangle$, where $E_{j}^{\prime }$ and $|\psi_{j}^{\prime }\rangle$ are the eigenenergies and eigenstates of *Ĥ*, and $c_{i,j}(t)=\langle \psi_{i}|\psi_{j}^{\prime }\rangle$. The probability of finding the system in a state |*ψ*_*k*_〉 of the measurement basis is

(A1) $$P_{k}(t)={\left|\langle \psi_{k}|\Psi (t)\rangle \right|}^{2}={\left|\sum_{i,j}a_{i}c_{i,j}c_{j,k}^{*}e^{-iE_{j}^{\prime }t/\hbar }\right|}^{2},$$

and can be written as a sum of oscillatory components

(A2) $$P_{k}(t)=1+\underset{i,j\neq l}{\sum }2\left|a_{i}^{2}c_{i,j}c_{j,k}c_{i,l}c_{l,k}\right|\text{cos}\left(2\pi f_{j,l}t\right),$$

where $f_{j,l}=\left(E_{j}^{\prime }-E_{l}^{\prime }\right)/h$ is the frequency associated with the energy difference of two eigenstates of the Hamiltonian.

Fourier spectroscopy relies on measuring the *P*_*k*_(*t*) as a function of time, and extracting the different frequency components *f*_*j*,*l*_ directly by computing the discrete Fourier transform. The bandwidth and frequency resolution of the measurement are determined by the total sampling time and the number of samples. For *N* samples separated by a time interval Δ*t*, the highest resolved frequency is *f*_bw_ = 1/2Δ*t* and the frequency resolution is Δ*f* = 1/Δ*tN*.

## APPENDIX B: SCATTERING RATE

Following the calculation of the scattering rate at the magic-zero wavelength in Refs. [43,44], we find that the scattering rate for a single beam with intensity *I*_0_ is

(B1) $$\gamma_{\text{sc}}=\frac{I_{0}\omega_{0}^{2}\alpha_{0}^{2}}{18\pi \epsilon_{0}^{2}\hbar^{3}c^{4}}{\left(\frac{1}{\Delta_{\text{D}1}^{2}}+\frac{2}{\Delta_{\text{D}2}^{2}}\right)}^{2},$$

where Δ_*D*1_ and Δ_*D*2_ are the detuning of the laser from the *D*1 and *D*2 lines (the 5*S*_1/2_ to 5*P*_1/2_ or to 5*P*_3/2_ transitions, respectively). The polarizability constant $\alpha_{0}=3\epsilon_{0}\hbar \lambda_{D2}^{3}\Gamma_{D2}/8\pi^{2}$, where *λ*_*D*2_ and Γ_*D*2_ are the resonant wavelength and natural linewidth of the *D*2 transition. Thus we have

(B2) $$\gamma_{\text{sc}}=0.00897{\text{s}}^{-1}\frac{I_{0}}{\left(\text{W}\;{\text{cm}}^{-2}\right)}.$$

For the two Raman beams here, we use *I*_0_ = *I*^+^ + *I*^−^, where $I^{-}=c\epsilon_{0}{\langle \left|E_{z}^{-}+E_{x}^{-}+E_{y}^{-}\right|\rangle }_{t}$, where ·〈·〉_*t*_ denotes the time average.

For all measurements described in the article, we have *ħ*Ω_*n*_ ≲ *E*_*R*_, which alone provides an upper bound on *I*_0_ and thus the photon scattering rate. We find *I*_0_ < 500W cm^−2^ and *γ*_sc_ < 4.5 s^−1^. Thus photon scattering does not hinder the utility of the Raman lattice presented here, as the scattering-limited lifetime is greater than 200 ms.

We confirmed this theoretical description by illuminating our BEC by the Raman lasers, but tuned far from two-photon resonance, and observed a ≈300 ms lifetime at intensities ≈2 higher than present in this experiment. We therefore confirm that the observed lifetime in our lattice is limited from technical considerations, most likely relative phase noise on any of the Raman fields, not the in-principle limit from spontaneous emission.
